# Primary pulmonary lymphoepithelioma-like carcinoma: A case report of pathological complete response (pCR) by neoadjuvant treatment

**DOI:** 10.1097/MD.0000000000024987

**Published:** 2021-03-19

**Authors:** Liqin Zhang, Tairan Hao, Yuqing Wei, Mo Dong, Yuancheng Xiong

**Affiliations:** The First Affiliated Hospital of Wannan Medical College, Yijishan Hospital, Wuhu, China.

**Keywords:** case report, chemotherapy, immunotherapy, pathological complete response, primary pulmonary lymphoepithelioma-like carcinoma

## Abstract

**Rationale:**

Primary pulmonary lymphoepithelioma-like carcinoma (PPLELC) is a rare subtype of non-small cell lung cancer (NSCLC). It is predominantly reported in East Asia and currently there is no standard treatment for this disease. We report a case of stage IV PPLELC that achieved pathological complete response (pCR) by neoadjuvant treatment.

**Patient concerns:**

The patient was a 46-year-old male who developed hemoptysis for about 20 ml of volume accompanied by cough and sputum after physical labor.

**Diagnoses:**

Contrast enhanced chest CT scanning showed occupation of left lower hilar area and left pleural effusion. Combined with medical history and auxiliary examination, the patient was formally diagnosed stage IV lymphoepithelioma-like carcinoma of the left lower lung (T3N0M1a pleura).

**Interventions:**

The patient was given Sintilimab combined with gemcitabine + nedaplatin chemotherapy (GP) regimen for four cycles with 3 weeks as a cycle, supplemented with antiemetics and stomach protection drugs to reduce chemotherapy-related side effects.

**Outcomes:**

After 4 cycles of treatment, the patient's left lung lesion has been markedly reduced and the left pleural effusion has also been significantly absorbed. Remarkably, surgical biopsies found no cancer cells in the lesion site and postoperative pathology showed complete pathological remission (pCR).

**Lessons:**

We reported a case of PPLELC that is sensitive to neoadjuvant treatment, showing excellent effectiveness and safety and achieving pCR.

## 1 Introduction

PPLELC is a rare subtype of non-small cell lung cancer (NSCLC)^[1]^ with different biological characteristics from other types of lung tumors and is composed of undifferentiated cancer cells, abundant lymphoid stroma, and cells with ultrastructural characteristics of squamous cell carcinoma. Although the etiology and pathogenesis of PPLELC are unclear, it is primarily seen in young non-smokers closely related to Epstein-Barr virus (EBV) infection and has significant ethnic and geographical distribution characteristics.^[2–4]^ Chest CT is commonly used for the diagnosis of PPLELC^[5]^ and pathological diagnosis and immunohistochemistry are generally used for the final diagnosis of PPLELC. At present, surgical treatment is the main treatment option for early stage PPLELC, and comprehensive treatment methods such as surgery, chemotherapy, and radiotherapy are often used for the middle and late disease stages.^[6–8]^

Most PPLELC patients lack mutations in EGFR and ALK, suggesting that targeted therapy may not provide significant benefits.^[9–12]^ Recent clinic studies demonstrated that programmed cell death ligand 1 (PD-L1) was highly expressed in PPLELC and patients with positive PD-L1 expression have better performance status and overall survival rates compared with those with negative PD-L1 expression when treated with PD-1/PD-L1 inhibitors.^[12–17]^ However, there are only few large-sample studies on the first-line treatment of PPLELC with PD-1/PD-L1 inhibitors while most of studies are small-sample second-line treatment case reports. Here we report a case of PPLELC that achieved pathological complete response (pCR) by neoadjuvant treatment combing immunotherapy with chemotherapy.

## 2 Case presentation

A 46-year-old male developed hemoptysis for about 20 ml of volume accompanied by cough and sputum after physical labor on February 4, 2020. Chest CT taken at a local hospital showed space-occupying lesion in the lung next to hilar of left lower lobe, accompanied by obstructive inflammation and left pleural effusion. The patient was admitted to the Respiratory and Critical Care Department of our hospital for further diagnosis and treatment. The patient had a history of smoking for 1 pack/day for 10 years but quitted smoking 3 years ago with no history of drug and food allergies. Physical examination showed no abnormalities except for low left lung breath sounds, dullness on percussion. Combined with medical history and auxiliary examination, preliminary diagnoses were:

1. Hemoptysis waiting for further investigation,
2. Space-occupying lesions of the left lower lung,
3. Obstructive pneumonia,
4. Left pleural effusion (small amount).

**Figure 1:**
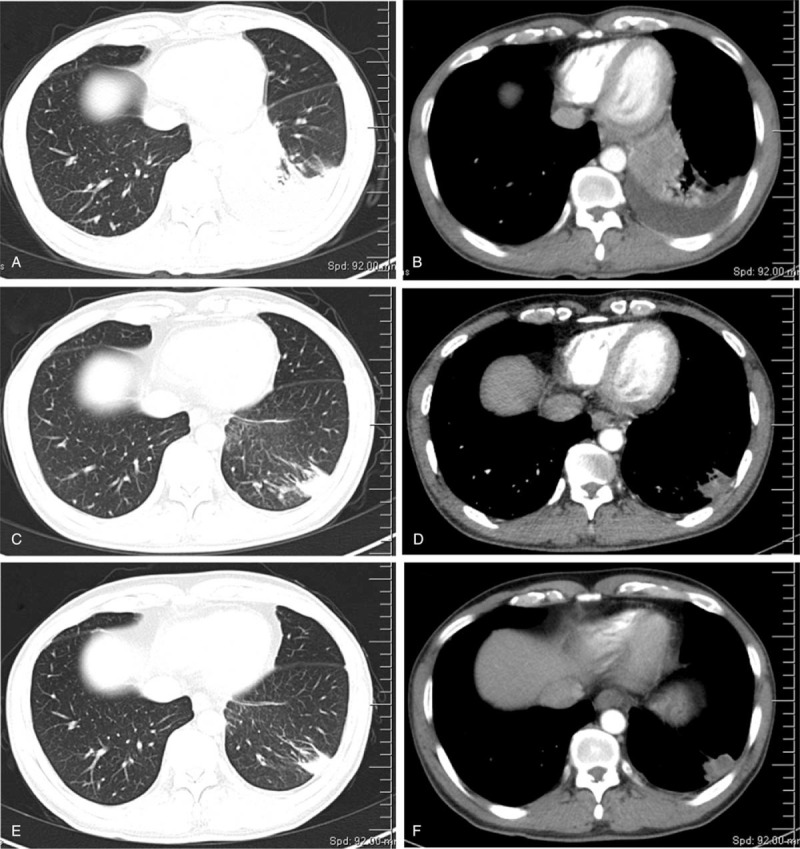
Chest CT scans of the patient before and after treatment. A-B: First chest CT scan taken on February 13, 2020, note the lesion and pleural effusion in the left lung; C-D: Reexamination of chest CT scan taken on April 20, 2020 after two cycles of treatment; E-F: Reexamination of chest CT taken on June 3, 2020 after four cycles of treatment.

After admission, results from ECG, heart color Doppler ultrasound, abdominal color Doppler ultrasound, and blood coagulation function appeared normal although Liver and kidney function tests suggests hypoproteinemia. All tumor markers tested were in normal ranges except levels of CA125 and CYFRA21-1 increased to 91.50u/ml and 5.31ng/ml, respectively. On February 13, 2020, contrast enhanced chest CT scanning showed:

1. Occupation of left lower hilar area, suggesting the possibility of central lung cancer and obstructive inflammation;
2. Left pleural effusion suggesting pleural invasion (Fig. 1A, B).

**Figure 2:**
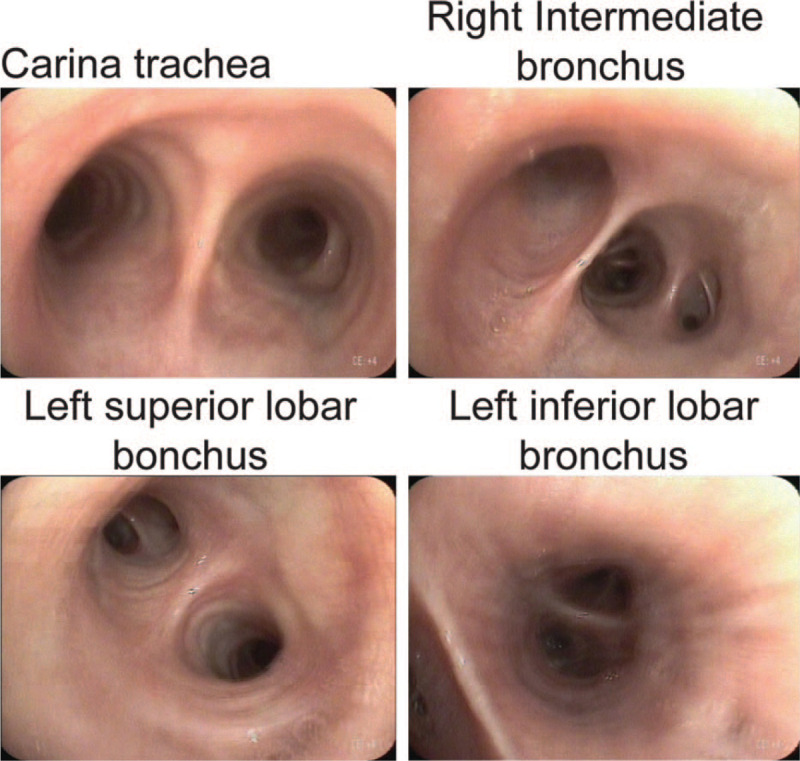
Electronic bronchoscopy examination. Representative images show that the lumen of each segment of the bronchus was unobstructed and the mucosa was smooth without bleeding or new growth.

Electronic bronchoscopy taken on February 12, 2020 showed that the lumens of the left and right bronchi were unobstructed without detectable new growth. The pathological smear of the posterior basal segment of the left lower lobe did not find malignant tumor cells (Fig. 2).

**Figure 3:**
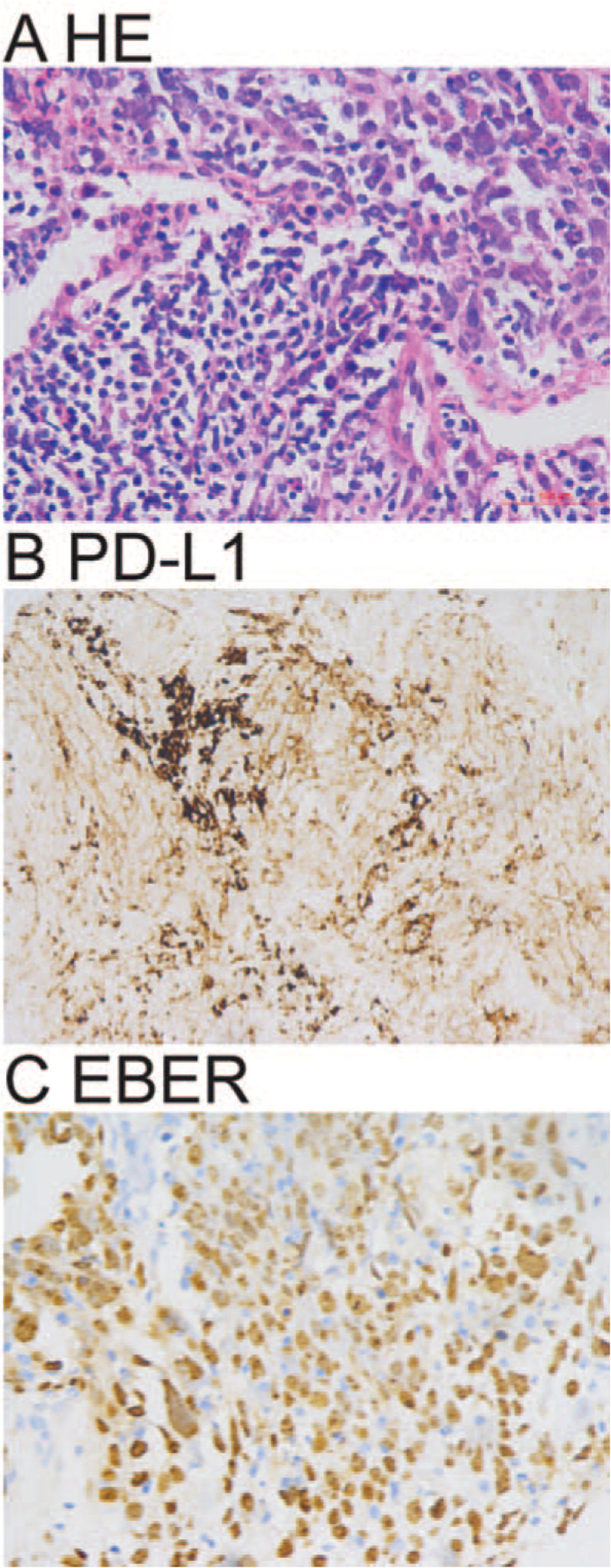
Percutaneous lung lesion biopsy examination. A. Representative histopathology of percutaneous lung lesion biopsy shows non-small cell carcinoma and lymphoepithelial carcinoma. B. Representative immunohistochemistry image shows PD-L1 (22C3) positive tumor cells. C: Representative in situ hybridization image shows EBER+ tumor cells. HE A × 400; Immunohistochemistry B × 400; in situ hybridization C × 400.

On February 14, CT-guided lung biopsy was obtained, confirming non-small cell carcinoma, lymphoepithelioma-like carcinoma (Fig. 3A). Immunohistochemistry showed: AE1/AE3 (+), TTF-1 (-), NapsinA (-), P63 (+), P40 (+), Ki-67 (30%, +); programmed cell death ligand 1 (Programmed cell death 1 ligand 1, PD-L1) (22C3). Tumor cell positive score was 55% (Fig. 3B) with positive EBV-encoded RNA (EBER+) (Fig. 3C). No abnormalities were found in head MRI scan + enhancement, whole body bone scan, and upper abdominal B-ultrasound. Genetic testing using the lung biopsy found no gene mutations in classic lung cancer driver genes *EGFR*, *ALK*, *K-RAS*, and *ROS1*. Based on above examinations and tests, the patient was formally diagnosed stage IV lymphoepithelioma-like carcinoma of the left lower lung (T3N0M1a pleura).

The patient and his family members signed the treatment consent form for cancer treatment after being fully informed of relevant examinations and baseline assessments, the risks of immunotherapy combined with chemotherapy and possible adverse reactions. Starting on February 29, 2020, the patient was given Sintilimab combined with gemcitabine + nedaplatin chemotherapy (GP) regimen for four cycles with 3 weeks as a cycle, supplemented with antiemetics and stomach protection drugs to reduce chemotherapy-related side effects. The most serious adverse reaction that the patient had experienced after the end of the second treatment cycle is grade IV bone marrow suppression, which was relieved after symptomatic treatment by stimulation of bone marrow hematopoietic function. On April 20, 2020, the follow-up chest CT showed:

1. Abnormal density and enhancement of the left lower hilar area became smaller compared to the CT enhancement results taken on February 13, 2020.
2. The lower lobe of the left lung had a patchy shadow, likely caused by local inflammation.
3. Thickening and adhesion of the left pleura (Fig. 1C, D).

After 4 cycles of treatment, the patient took another chest CT on June 3, 2020 and found that there was a nodular high-density shadow in the left lower hilar area, about 1.5cm∗1.3 cm in size (Fig. 1E, F), which is smaller than that found in the chest CT scan taken on April 20, 2020. Considering the fact that the patient's left lung lesion has been significantly reduced in the reexamination, and the left pleural effusion has also been significantly absorbed, our department consulted a lung cancer multi-disciplinary team which agreed that the patient was degraded by stage and recommended surgical treatment followed by chemotherapy and immunotherapy based on surgical pathology and staging after surgery.

**Figure 4:**
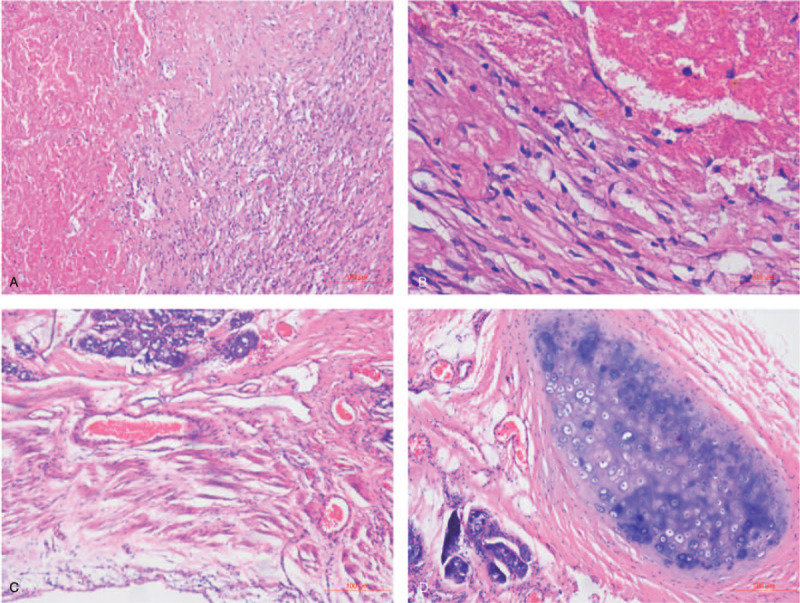
Postsurgery pathological examination. A, B: There were no cancer cells found in the sections of lower left lung lesion site. C, D: Intraoperative frozen section pathology showed that there was no tumor component in the bronchial stump. HE A × 100, B × 400, C × 100, D × 100.

The patient underwent single port VATS left lower lobectomy + lymph node dissection in our hospital under general anesthesia at the Department of Thoracic and Cardiovascular Surgery on June 8, 2020. After surgery, the left lower lung, group 4L lymph node, and group 5 to 11 lymph nodes were sent for pathological inspection. Solitary necrotic nodule of the left lower lung (peripheral type, about 3.5 cm in diameter) and collagenized nodule with lymphocyte infiltration and focal area calcification (central type, about 1.8 cm in diameter) were reported, which were considered as an inflammatory response to chemotherapy. No cancer cells were found at the bronchial resection margin (Fig. 4A and B). Moreover, intraoperative frozen section pathology found no tumor component in the bronchial stump (Fig. 4C and D). Thus, the patient's postoperative pathology showed complete pathological remission (pCR), and he was discharged from hospital after a full recovery from the operation and was followed up regularly.

**Figure 5:**
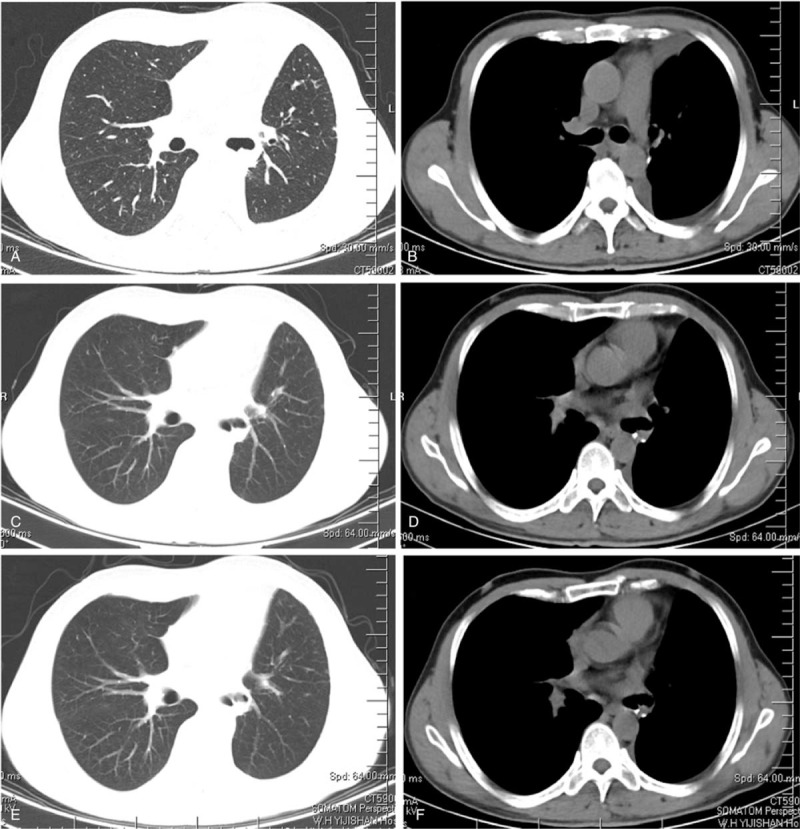
Postsurgery follow-up chest CT scans. A, B: The first follow-up chest CT after surgery was taken on June 24, 2020. C, D: The second follow-up postoperative chest CT scan was taken on September 2, 2020. E, F: The third follow-up postoperative chest CT scan was taken on November 4, 2020.

The patient's chest CT was retaken on June 24, 2020 (Fig. 5A, B), September 2, 2020 (Fig. 5C, D), and November 4, 2020 (Fig. 5E, F). All his chest CT scans showed complete remission (CR). Up to November 4, 2020, the patient is in good condition and the blood tumor markers, liver, gallbladder, pancreas, spleen and abdomen were all normal in his outpatient examination.

## 3 Discussion

PPLELC was first reported by Begin in 1987.^[1]^ In the latest 2015 WHO lung cancer histopathological classification, PPLELC was classified into other unclassified cancer group, reflecting that PPLELC has different biological characteristics from other types of lung tumors. The etiology and pathogenesis of PPLELC are unclear. Similar to nasopharyngeal carcinoma, PPLELC is composed of undifferentiated cancer cells, abundant lymphoid stroma, and cells with ultrastructural characteristics of squamous cell carcinoma.

PPLELC can be located under the pleura or beside the mediastinum and displays round and lobulated masses, most of which have smooth edges and clear borders while pleural traction and burr signs are rare seen in chest CT scan. Plain scan often shows low-density patchy necrosis area and enhanced scan reveals uneven and moderately clear enhancement, showing vascular or bronchial encasement.^[5]^ PPLELC can also partially surround the nearby bronchus and blood vessels. Pathological diagnosis and immunohistochemistry often find abundant lymphocytes beside tumor cells, and lymphoid follicle formation is also an important feature. When necessary, nasopharyngoscopy should be performed to exclude lymphoepithelioma-like carcinoma metastasis in other parts of the nasopharynx.

Most PPLELC patients lack mutations in common tumor promoting genes such as *EGFR* and *ALK*.^[9–11]^ In this case, no mutations were found in the classic driver genes such as *EGFR*, *ALK*, *K-RAS*, and *ROS1*. Recent exciting basic and clinic studies demonstrated that immunotherapy and immunotherapy improve the immune function of the body, increase the activity of immune cells, and reduce the suppression of immune cells by the tumor microenvironment. Recent clinic studies have also demonstrated that PD-L1 was highly expressed in the tumor cells from PPLELC patients, and patients with positive PD-L1 expression have better performance status (PS) and overall survival rates compared with those with negative PD-L1 expression when treated with PD-1/PD-L1 inhibitors.^[12–15]^ However, at present, there are only few large-sample studies on the first-line treatment of PPLELC with PD-1/PD-L1 inhibitors, and most of them are second-line treatment case reports and small-sample studies. Moreover, if immunotherapy can be used as the new standard therapy for advanced PPLELC remains unclear.

Currently, there are 3 neoadjuvant immunotherapy treatment options:

1. Neoadjuvant immunotherapy→surgical treatment;
2. Neoadjuvant immunotherapy + chemotherapy→surgical treatment;
3. Neoadjuvant immunotherapy→surgical treatment→postoperative adjuvant immunotherapy.

So far, there are no follow-up treatments of neoadjuvant immunotherapy combined with chemotherapy for the PPELEC patients, although some small randomized controlled clinical studies of neoadjuvant immunotherapy have been reported.^[17]^ Sintilimab is a PD-1 inhibitor currently undergoing a number of phase I, phase II, and phase III clinical trials in China. The ORIENT-11 study^[16,18]^ is a phase III clinical trial using Sintilimab combined with pemetrexed and platinum as the first-line treatment of non-squamous NSCLC. This study shows that compared with chemotherapy alone, first-line use of sintilimab combined with chemotherapy for advanced/recurrent non-squamous NSCLC without EGFR+ mutations or *ALK* gene rearrangement has significant benefits. In our case, the patient's tumor cell positive score is 55%, suggesting a high PD-L1 expression. Therefore, based on the patient's specific economic status, combined with the efficiency of the GP program in previous studies, the final personalized treatment plan for this patient was: PD-1 inhibitor (sintilimab) combined with chemotherapy (gemcitabine + nedaplatin). After 4 cycles of immunotherapy and chemotherapy, the lesion was markedly reduced.

In this case, after 4 cycles of neoadjuvant sintilimab immunotherapy combined with GP chemotherapy, the lesion was significantly smaller than before and postsurgical histopathology showed no cancer cells, and therefore the therapeutic effect reached pCR. Although it was recommended that patients took Sintilimab maintenance treatment for 1 year to prevent recurrence, the patient agreed to cooperate with the follow-up visits but refused further Sintilimab treatment. The examinations have been normal 5 months after the operation and the patient is currently being followed up regularly.

In summary, PPLELC is a rare subtype of NSCLC, and currently there is no standardized treatment. This study reported a case of elective surgical resection for PPLELC after 4 cycles of neoadjuvant sintilimab immunotherapy combined with GP regimen chemotherapy. This patient was well tolerated during neoadjuvant combined therapy, and the lesion was significantly reduced. Surgical resection confirmed pCR, and the patient's quality of life has been significantly improved. Our case suggests that neoadjuvant treatment alone can be sufficient to achieve pCR of PPLELC with high PD-L1 expression and argue that posttreatment surgery might not be necessary for PPLELC patients.^[19]^ However, the effectiveness, safety, drug dosage, and adverse drug reactions of this treatment program need to be further explored and confirmed in PPLELC patients with high PD-L1 expression.

## 4 Disclosure

This study was approved by the institutional review board of the The First Affiliated Hospital of Wannan Medical College, Yijishan Hospital. The requirement for informed consent was obtained from the patient and his family members. There is no conflict of interest exists in the submission of this manuscript, and manuscript is approved by all authors for publication.

## Author contributions

**Conceptualization:** Liqin Zhang.

**Data curation:** Tairan Hao, Yuqing Wei, Mo Dong, Yuancheng Xiong.

**Formal analysis:** Tairan Hao.

**Investigation:** Tairan Hao.

**Writing – original draft:** Liqin Zhang, Tairan Hao, Yuqing Wei, Mo Dong, Yuancheng Xiong.

**Writing – review & editing:** Liqin Zhang.
